# Assessment and Prognosis in CSA-AKI Using Novel Kidney Injury Biomarkers: A Prospective Observational Study

**DOI:** 10.3390/biology10090823

**Published:** 2021-08-24

**Authors:** Jakub Udzik, Aleksandra Waszczyk, Krzysztof Safranow, Andrzej Biskupski, Krzysztof Majer, Sebastian Kwiatkowski, Ewa Kwiatkowska

**Affiliations:** 1 Department of Cardiac Surgery, Pomeranian Medical University, PowstańcówWielkopolskich 72, 70-111 Szczecin, Poland; a.biskupski@vp.pl (A.B.); krzysztofmajer88@gmail.com (K.M.); 2 Department of Infectious Diseases, Hepatology and Liver Transplantation, Pomeranian Medical University, Arkońska 4, 71-455 Szczecin, Poland; 1603aleksandra@gmail.com; 3 Department of Biochemistry and Medical Chemistry, Pomeranian Medical University, PowstańcówWielkopolskich 72, 70-111 Szczecin, Poland; chrissaf@mp.pl; 4 Department of Obstetrics and Gynecology, Pomeranian Medical University, PowstańcówWielkopolskich 72, 70-111 Szczecin, Poland; kwiatkowskiseba@gmail.com; 5 Department of Nephrology, Transplantology and Internal Medicine, Pomeranian Medical University, PowstańcówWielkopolskich 72, 70-111 Szczecin, Poland; ewakwiat@gmail.com

**Keywords:** CSA-AKI, novel kidney injury biomarkers, cardiac surgery, cardiopulmonary bypass, intraoperative hemofiltration

## Abstract

**Simple Summary:**

Cardiac surgery associated acute kidney injury is a common complication of cardiac surgeries that worsens postoperative outcomes and the patient’s prognosis. One of the main problems concerning this complication is its early diagnostics. In this study, the authors attempt to provide new data regarding the usage of novel kidney injury biomarkers in early detection of acute kidney injury and in the prognosis of the long-term postoperative kidney function. Perioperative factors that may influence kidney function were also analysed. It was concluded that biomarkers such as IL-6, IL-8, TNF-α, MMP-9 and NGAL are reliable acute kidney injury indicators. It was also demonstrated that intraoperative hypoperfusion secondary to hypovolemia is a main factor that damages the kidneys during cardiac surgeries. The authors hope that these findings will contribute to the development of new diagnostic protocols for acute kidney injury, which would involve novel renal biomarkers, thus enabling quicker diagnosis and more effective treatment of acute kidney injury.

**Abstract:**

Background: There is a need for early diagnostic solutions for cardiac surgery associated acute kidney injury (CSA-AKI) as serum creatinine changes do not occur dynamically enough. Moreover, new approaches are needed for kidney protective strategy in patients undergoing cardiac surgery procedures; Methods: Samples of serum and urine were taken from the selected group of patients undergoing elective cardiac surgery procedures. The aim of this study was to assess the utility of specific inflammation and kidney injury biomarkers in the early diagnostic of CSA-AKI and in the prognosis of long-term postoperative kidney function; Results: At 6 h after weaning from cardiopulmonary bypass, there were significant differences in IL-6, IL-8, TNF-α, MMP-9 and NGAL concentrations in patients with CSA-AKI, compared to the control group. Serum IL-8 and urine NGAL 6 h after weaning from CPB proved to be independent acute kidney injury predictors. The TNF-α, MMP-9, IL-18, TIMP-1 and MMP-9/TIMP-1 ratio in the early postoperative period correlated with long-term kidney function impairment; Conclusions: Novel kidney injury biomarkers are an eligible tool for early diagnosis of CSA-AKI. They are also reliable indicators of long-term postoperative kidney function impairment risk after cardiac surgery procedures.

## 1. Introduction

Cardiac surgery procedures are related to a high incidence of nephrological complications. These complications concern 5–30% of patients who undergo heart surgery, increasing mortality in this group of patients even up to 80% [1]. The use of cardiopulmonary bypass (CPB) is a factor that most probably contributes to postoperative kidney function impairment, but its role in the development of cardiac surgery associated acute kidney injury (CSA-AKI) is still unclear [2]. CPB may cause kidney damage in three main mechanisms: microcirculation disruption, systemic inflammatory response and increased oxidative stress [3]. Proinflammatory cytokines play a considerable role in these mechanisms of kidney damage. Propagation of the immune response takes place while blood leukocytes make contact with the artificial surface of the CPB tubing system-role of interleukin 6 (IL-6), interleukin 8 (IL-8) and tumor necrosis factor alpha (TNF-α). Both enhanced immune response and increased oxidative stress (secondary to extracorporeal oxygenation) intensify microcirculation disruptions in the renal tubules arterioles, leading to ischemia within these structures.

eGFR, which derives from serum creatinine concentration (S_Cr_), is a classical parameter used for kidney function monitoring. It is, however, an indirect indicator of kidney damage which demonstrates only the loss of function. AKI biomarkers such as kidney injury molecule 1 (KIM-1), neutrophil gelatinase-associated lipocalin (NGAL) and interleukin 18 (IL-18) are direct and far more specific indicators of kidney damage. Many scientific reports were published that state their usefulness in the diagnostic of AKI, including CSA-AKI [4,5,6].

Both NGAL and KIM-1 are highly specific kidney injury biomarkers, which are produced by the structures of the nephron in response to its damage [7,8]. NGAL appears in urine after 3 h from kidney injury, peaks at 6 h and maintains elevated for a longer time period stimulating tissue regeneration [6]. According to some authors, persistently elevated urine NGAL is an independent risk factor for chronic kidney disease (CKD) development [9]. KIM-1 appears in urine several h after a damage to the nephron structures, and in 2–6 h after weaning from CPB, it has 90% sensitivity in detecting CSA-AKI [10]. Urine KIM-1 peaks at 48–72 h after the injury [5], and it stays elevated until proximal tubules are completely regenerated. Similarly to NGAL, it has a protective effect on kidney cells by enhancing their regeneration. Persistent urine KIM-1 elevation is also a risk factor for CKD [11].

Urine IL-18 increases 4–6 h after a cardiac surgery procedure with the use of CPB, peaks at 12 h and normalizes in 48 h [12,13]. A meta-analysis of the previously conducted research showed that it has 58% sensitivity and 75% specifity in detecting CSA-AKI [14].

Moreover, cytokines such as IL-6, IL-8, TNF-α, matrix metalloproteinase 9 (MMP-9) and tissue inhibitor of metalloproteinase 1 (TIMP-1) are used for inflammatory response and organ damage assessment, including AKI [15,16,17,18]. IL-6 and IL-8 are both sensitive biomarkers of inflammatory processes. Their serum concentrations elevate significantly in patients with CSA-AKI as early as 2–12 h after weaning from CPB. Monitoring the increase in serum IL-6 and IL-8 in critically ill patients with AKI is important, because it is related to higher mortality risk during hospitalization [19].

TNF-α plays a major role in the pathophysiology of ischemia-reperfusion injury—IRI, which is one of the basic mechanisms underlying AKI [20]. For this reason, it is used in scientific studies to diagnose CSA-AKI. Its serum concentration is significantly higher 6 h after the operation in patients with AKI compared to the control group [21].

MMP-9 and its tissue inhibitor TIMP-1 play a key role in extracellular matrix (ECM) remodeling, including remodeling secondary to injury. Of diagnostic significance are not only MMP-9 and TIMP-1 concentrations, but also their mutual relation—MMP-9/TIMP-1 ratio [16,18]. Elevated MMP-9 serum concentration can be observed as early as several h after the damaging factor appears, whereas TIMP-1 serum concentration peaks at 24–48 h after the injury [22]. Serum concentrations of MMP-9 and TIMP-1 correspond well with their urine concentration, even in case of impaired kidney function [23,24].

Intraoperative hemofiltration is a procedure mainly used to remove water from the vascular bed in case of excessive hemodilution associated with CPB. Some researchers have also found that intraoperative hemofiltration can reduce serum proinflammatory cytokines concentration [25,26]. In the current study, authors decided to investigate this lead further and see if the use of hemofiltration can result in lower postoperative cytokines concentration and better outcomes of the procedure.

AKI is a serious clinical condition, and the time to make a diagnosis is vital in terms of treatment results [27]. Three basic scales are used to diagnose AKI—Table 1.

Demonstrated criteria of AKI diagnosis are based on a serum creatinine increase and diuresis, which are both indirect kidney damage indicators. Serum creatinine concentration after a cardiac surgery procedure with the use of CPB may be inadequately low due to high fluids administration or intraoperative hemofiltration. Decreased diuresis may also result from intraoperative hemofiltration or from prerenal causes such as low cardiac output syndrome (LCOS) which is a frequent complication of heart surgeries [28], but does not necessarily lead to AKI.

Regarding the above, using serum creatinine concentration and diuresis for detecting CSA-AKI may extend the time to make this diagnosis and apply suitable treatment. CSA-AKI is a specific type of AKI, as the physician knows exactly when it may occur. The problem lies in early identification of patients in whom the surgery was complicated by AKI. For this reason, the aim of this study was to assess the utility of specific inflammation and kidney injury biomarkers (IL-6, IL-8, TNF-α, NGAL, KIM-1, IL-18, MMP-9 and TIMP-1) in the early diagnostic of CSA-AKI and in the prognosis of long-term postoperative kidney function. The assessment also included perioperative factors that might influence postoperative kidney function.

## 2. Materials and Methods

**Scheme 1:**
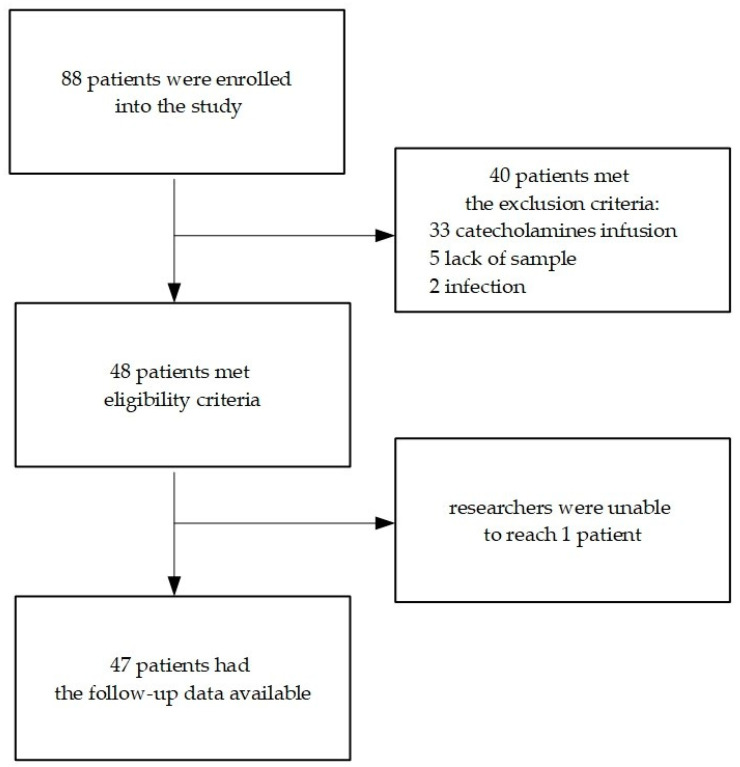
Patients enrollment into the study.

A total of 88 patients in the cardiac surgery clinic (31 women and 57 men) who qualified for an elective cardiac surgery procedure with the use of CPB were initially enrolled into the study. Patients’ medical history, laboratory results and postoperative outcomes were analysed. A total of 48 patients (18 women and 30 men) were eventually included in the study—Scheme 1.

The most frequent reason for a patient’s exclusion proved to be prolonged catecholamines infusion (33 patients—82.5% of excluded group). Less frequent reasons included a lack of sample on the 5th day after the operation (5 patients—12.5% of excluded group) and early postoperative infection (2 patients—5% of the excluded group; one patient developed postoperative pneumonia, and the other had postoperative wound infection). Follow-up data were obtained from 47 patients (compliance rate was 97.92%).

Samples of blood and urine were taken at the designated time points (Table 2). Preoperative samples were collected in the operating room directly after the induction of general anesthesia. Should the patients require continuous renal replacement therapy (CRRT) after the operation, an additional blood sample was to be taken 6 h after the end of treatment (S2). Eventually, none of the patients from this study population required renal replacement therapy after the operation; thus, the “S2” blood sample was not taken from any patient.

Blood was collected using S-Monovette 3.4 mL sterile containers (K3 EDTA: 1.6 mg/1 mL of blood; SARSTEDT AG & Co. KG Sarstedtstrasse 1, 51588 Nümbrecht, Germany). Urine was collected using standard non-sterile urine containers. After the collection, samples were stored at 5 °C for no longer than 4 h and subsequently centrifuged (4 °C, 10 min, 4000 RPM). After centrifugation, 1 mL of supernatant was taken and stored at −70 °C.

In patients eventually included in the study, long-term postoperative kidney function was assessed after no less than 3 months from the date of the operation, with accordance to KDIGO guidelines [29]. For this purpose, serum creatinine concentration was measured, and the estimated glomerular filtration rate (eGFR) was subsequently calculated using both CKD-EPI and MDRD formulas for each patient. Preoperative eGFR and eGFR in the early postoperative period were calculated using the CKD-EPI formula.

Inclusion criteria:

- obtaining a written consent for study enrollment
- qualification for elective cardiac surgery procedure with the use of CPB, age > 18 years.

Exclusion criteria:

- CKD stage V according to KDIGO (eGFR < 15 mL/min/1.73 m^2^)
- taking nephrotoxic medications before the operation (including loop diuretics)
- degenerative changes in renal arteries in medical history
- active neoplasm disease
- catecholamines administration longer than 3 h after the procedure or new catecholamines administration after more than 3 h after the procedure
- postoperative infection (up to 7 days after the procedure)
- cardiac arrest in the postoperative period
- early decease (up to 7 days after the procedure)
- lack of any of the samples from Table 2

**Scheme 2:**
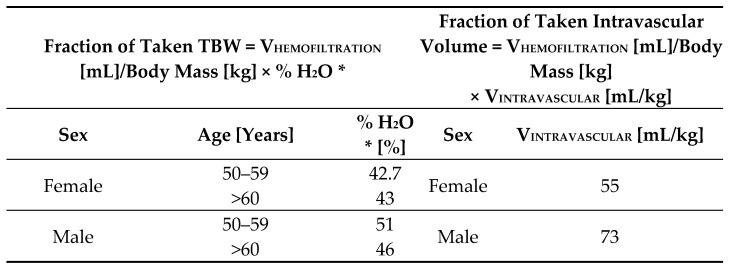
Formulas used to calculate fraction of taken TBW and intravascular volume. Legend: TBW—total body water [mL], V_HEMOFILTRATION_—hemofiltration volume [mL], V_INTRAVASCULAR_—intravascular volume [mL/kg].

In order to adjust the hemofiltration volume for patient’s sex and body mass, a percentage of taken total body water (TBW) was calculated for each patient who underwent intraoperative hemofiltration (based on the publication by Chumlea et al. [30]). The percentage of water taken from the intravascular space was also calculated for these patients (based on the publication by Feldschuh et al. [31]). Formulas are depicted in Scheme 2.

Intraoperative hematocrit level was measured 10–15 min after the initiation of CPB (Ht_CPB_1) and subsequently every 30 min during the whole CPB procedure (Ht_CPB_2 etc.). Intraoperative hemofiltration was initiated basing on a decision of the operating team (cardiac surgeon, anesthesiologist, perfusionist) with regard to the patient’s condition and CPB parameters. Main reasons for implementing this procedure were hematocrit level < 22% and intraoperative anuria.

Postoperative creatine kinase MB isoenzyme (CK-MB) was measured in the 6th, 9th and 12th h after the operation. Postoperative creatinine and C-reactive protein (CRP) concentrations were measured on the 1st day after the operation and subsequently every 48 h for the next 5 days (in some patients, measurements were also taken on the 7th day). During the first 24 h after the operation, diuresis was measured every 2 h.

Patients were monitored for AKI development for 5 consecutive days after the operation. The KDIGO scale was assumed as a referential scale for diagnosing AKI. The simultaneous assessment of kidney function was performed using RIFLE and AKIN criteria.

In patients included in the study, a total dose of catecholamines was noted, including both intraoperative and postoperative catecholamines administration. The dose was then adjusted for patient’s body mass (mg/kg of body mass).

The study population was divided into study and control groups based on the following end points: AKI development during the first 5 days after the operation according to KDIGO criteria, long-term postoperative kidney function impairment (defined as eGFR decline after ≥3 months after the operation < 60 mL/min/1.73 m^2^ in patients with preoperative eGFR > 60 mL/min/1.73 m^2^ or eGFR decline in patients with preoperative eGFR < 60 mL/min/1.73 m^2^ that qualifies them into the next CKD stage) and undergoing intraoperative hemofiltration.

Quantitative measurements of serum IL-6, IL-8 and TNF-α and urine NGAL, KIM-1, IL-18 and MMP-9 in patients included in the study were performed using the Luminex technology. The method involved magnetic microspheres with a solid phase for antibodies or antigens immobilized on their surface. A 2-fold dilution was applied to urine samples, whereas serum samples were not diluted. Commercial Luminex Human Discovery Assays (R&D Systems, Minneapolis, MN, USA) were used to measure IL-6, IL-8, TNF-α, NGAL, KIM-1, IL-18 and MMP-9 concentrations. The quantification procedure was performed according to manufacturer’s instruction using Luminex 200 device (Luminex Corporation, Austin, TX, USA). Reagents’ concentrations were calculated using a standard 6-points curve.

Total urine concentrations of TIMP-1 were quantified using an enzyme-linked immunosorbent assay (ELISA) kit (Quantikene; R&D Systems, Inc., Minneapolis, MN, USA), performed according to manufacturer’s instructions.

Most of the quantitative data in this study had distributions considerably different from the normal distribution (Shapiro–Wilk test). Hence, non-parametric tests were applied to analyze that data while median and quartiles were used for descriptive statistics: M, (Q1-Q3). The U Mann–Whitney test was used to compare data between the groups, and the assessment of changes’ significance was performed using Friedman’s ANOVA test and Wilcoxon signed-rank test. Correlations between the variables were analyzed using the Spearman rank correlation coefficient (Rs). Qualitative data were compared between the groups using a chi-square test or Fisher’s exact test for 2 × 2 tables. In order to determine independent AKI predictors, multiple logistic regression analysis was performed. A statistical significance level of *p* < 0.05 was assumed. Statistical analysis was conducted using licensed Statistica 13.6.0 software (StatSoft, Inc., Tulsa, OK, USA). 

The dataset generated for this study is available as Supplementary Material—Table S1.

## 3. Results

Mean time from the operation to the control creatinine measurement was 34 ± 14 weeks, and the median was 37 weeks; the shortest follow-up time was 13 weeks, and the longest was 66 weeks. Baseline characteristics and operation-related data of all patients initially enrolled into the study are presented in Table 3. Data of patients eventually included in the study and the excluded ones were compared. Aside from the presence of exclusion criteria, there were no significant differences between the groups.

### 3.1. AKI vs. no-AKI

CSA-AKI developed in 15 patients (eight women and seven men), which accounts for 31.25% of the study population. AKI was most frequently diagnosed on the 1st day after the operation (66.67%), and the latest diagnosis was made on the 3rd postoperative day (13.33%). Patients who developed postoperative AKI were older (M = 70 (67–79) years vs. 66 (61–70) years in the control group, *p* = 0.013), had worse preoperative kidney function (creatinine concentration: M = 1.03 (0.9–1.28) mg/dL vs. 0.88 (0.78–0.99) mg/dL in the control group, *p* = 0.012; eGFR value: M = 62 (53–75) mL/min/1.73 m^2^ vs. 85 (78–93) mL/min/1.73 m^2^ in the control group, *p* < 0.001), higher preoperative CKD incidence (40% vs. 6.06% in the control group, *p* = 0.008) and lower preoperative hematocrit (M = 37.8 (35.5–40.6)% vs. 40.9 (38.6–44.1)% in the control group, *p* = 0.002). There were no significant differences in arterial hypertension, diabetes or dyslipidemia incidence between the groups.

Regarding intraoperative features: patients with AKI were administered a higher amount of intravenous fluids during the operation (M = 3500 (2800–3850) mL vs. 2800 (2400–3300) mL in the control group, *p* = 0.016), had lower intraoperative diuresis (M = 800 (300–1500) mL vs. 1600 (1200–1900) mL in the control group, *p* = 0.006), and as a result higher fluid balance from the time of the operation (M = 700 (300–1200) mL vs. −100 (–400–500) mL in the control group, *p* < 0.001). These patients also had a lower hematocrit level during CPB (Ht_CPB_1: M = 20 (19–22)% vs. 23 (19–25)% in the control group, *p* = 0.012; Ht_CPB_2: M = 23.5 (21–27)% vs. 27.5 (25.5–29.5)% in the control group, *p* = 0.004) and a higher percentage of patients undergoing coronary artery bypassing graft (CABG) combined with the valvular procedure (CABG + valvular: 33.33% vs. 3.03% in the control group, *p* = 0.008), although there were no significant differences in aortic cross-clamp time or CPB duration time (*p* = 1.000 in both cases).

Patients with AKI had lower diuresis during the first 2 h after the procedure (M = 300 (200–800) mL vs. 800 (400–1000) mL in the control group, *p* = 0.040).

**Figure 1:**
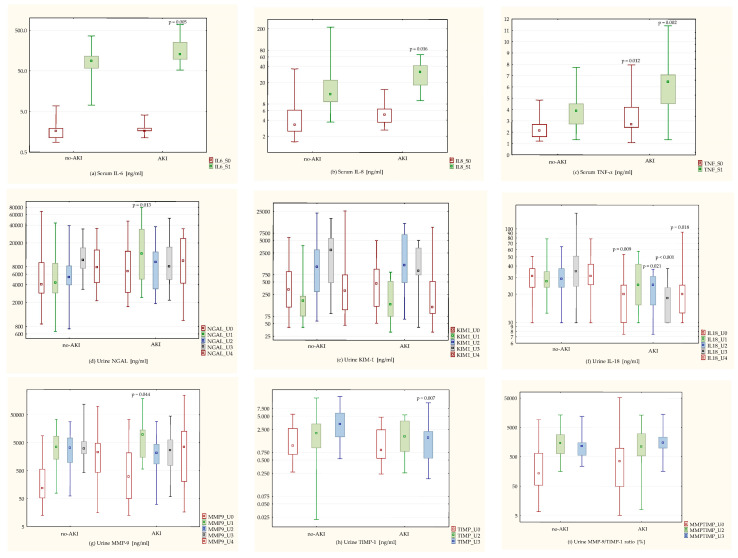
Biomarkers’ concentrations (**a**–**i**) at the designated time points in patients without AKI vs. with AKI. Sampling time descriptors in the legend (S0, S1, U0–U4) are consistent with the time points mentioned in Table 2. Dots on the chart represent the median value; boxes represent values between the 1st and 3rd quartile (Q1–Q3), and whiskers represent the extreme values. For values that differed significantly between the study and control group (*p* < 0.05), *p*-values are written above the upper whisker in the AKI group.

Biomarkers’ concentrations in the AKI group (Figure 1) were as follows: higher preoperative serum TNF-α (M = 2.7 (2.42–4.22) ng/mL vs. 2.13 (1.59–2.7) ng/mL in the control group, *p* = 0.012) and lower preoperative urine IL-18 (M = 19.93 (9.88–25.06) ng/mL vs. 31.05 (23.52–37.62) ng/mL in the control group, *p* = 0.009). At 6 h after weaning from CPB, patients with AKI had higher serum IL-6 (M = 128.58 (95.53–255.2) ng/mL vs. 86.62 (57.36–115.84) ng/mL in the control group, *p* = 0.005), IL-8 (M = 31.02 (17.89–41.66) ng/mL vs. 12.09 (8.74–22.27) ng/mL in the control group, *p* = 0.016) and TNF-α (M = 6.43 (4.5–7.08) ng/mL vs. 3.9 (2.7–4.52) ng/mL in the control group, *p* = 0.002). Urine concentrations of NGAL and MMP-9 were also significantly higher in these patients (NGAL: M = 13231.76 (4914.81–34148.3) ng/mL vs. 4261.32 (2868.19–9138.28) ng/mL in the control group, *p* = 0.013; MMP-9: M = 9661.94 (1485.62–14,451.8) ng/mL vs. 3499.39 (1274.58–8656) ng/mL in the control group, *p* = 0.044). Absolute urine IL-18 concentration 6 h after weaning from CPB did not differ significantly between the groups (*p* = 0.597), but in patients with AKI, there was a significant increase in urine IL-18 compared to the initial value (M = 122.02 (100–204.21)% vs. 89.8 (77.97–112.53)% in the control group, *p* = 0.002). Urine TIMP-1 concentration 48 h after the operation was significantly lower in patients with AKI, regarding both the absolute values (M = 1.61 (0.55–2.26) ng/mL vs. 3.3 (1.68–5.92) ng/mL in the control group, *p* = 0.007) and the percentage of increase from the initial value (M = 145.05 (64.91–211.91)% vs. 268.81 (111.43–733.73)% in the control group, *p* = 0.030).

Nine patients in the AKI group underwent intraoperative hemofiltration (60% of the AKI group) vs. nine patients who underwent intraoperative hemofiltration in the no-AKI group (27.27%). The difference, however, was not statistically significant (*p* = 0.052).

Comparing to KDIGO criteria, the RIFLE scale demonstrated 53.33% sensitivity and 100% specificity in diagnosing AKI. The AKIN scale had 100% sensitivity and 100% specificity in diagnosing AKI when compared to KDIGO criteria.

The diuresis criterion (<0.5 mL/kg/h for at least 6 h) allowed the diagnosis of AKI in one patient in the 22nd hour after the operation (6.67% of patients with CSA-AKI).

Multiple logistic regression analysis adjusted for patients’ age proved that the best independent predictors of CSA-AKI are: intraoperative diuresis (OR = 0.047, CI: 0.005–0.451 over 1000 mL, *p* = 0.006), IL-8 6 h after weaning from CPB (OR = 11.991, CI: 1.549–92.830 over 1 ng/mL, *p* = 0.014) and NGAL 6 h after weaning from CPB (OR = 3.434, CI: 1.180–9.991 over 1 ng/mL, *p* = 0.020).

### 3.2. Long-Term Postoperative Kidney Function Impairment vs. Unchanged Long-Term Postoperative Kidney Function

In the long-term observation (≥3 months after the procedure), kidney function impairment was observed in four patients (three women and one man), which accounts for 8.33% of the study population. Three patients developed CKD (two patients in KDIGO stage 3a and one patient in KDIGO stage 3b) subsequently to developing postoperative AKI. In one patient who did not suffer from CSA-AKI, progression of a preoperative CKD was observed (from KDIGO 3a stage to KDIGO 3b stage).

Patients in whom long-term postoperative kidney function impairment was observed had lower preoperative eGFR (M = 61 (55–68.5) mL/min/1.73 m^2^ vs. 82 (68.5–91) mL/min/1.73 m^2^ in the control group, *p* = 0.035), higher mean hemofiltration volume (M = 1500 (750–1750) mL vs. 0 (0–900) mL in the control group, *p* = 0.039), higher percentage of taken TBW (M = 5.2 (3–6.64)% vs. 0 (0–2.71)% in the control group, *p* = 0.028), higher percentage of water taken from the intravascular volume (M = 40.56 (17.05–51.92)% vs. 0 (0–16.62)% in the control group, *p* = 0.028) and lower diuresis between 2nd and 4th hour after the procedure (M = 150 (75–200) mL vs. 400 (200–800) mL in the control group, *p* = 0.009).

Patients with long-term postoperative kidney function impairment did not differ significantly from the patients in a control group in terms of arterial hypertension, diabetes, dyslipidemia and preoperative CKD incidence. There was also no difference between the types of procedures performed in both groups.

Intraoperative hemofiltration was used in three patients with long-term postoperative kidney function impairment (75% of the group), compared to 15 patients in whom long-term postoperative kidney function did not change (34.09% of the group). The difference was not statistically significant (*p* = 0.142).

There was a strong correlation between preoperative kidney function and kidney function in a long-term observation. A similar correlation was observed between early postoperative kidney function and long-term kidney function—Table 4.

There was a negative correlation between serum TNF-α concentration and eGFR value after ≥3 months from the operation (TNF-α & eGFR by CKD-EPI: r = −0.431, *p* = 0.002; TNF-α & eGFR by MDRD: r = −0.361, *p* = 0.013), whereas for serum TNF-α concentration 6 h after weaning from CPB, a significant correlation was only with eGFR by CKD-EPI after ≥3 months (r = −0.372, *p* = 0.01).

Urine IL-18 concentration 6 h after weaning from CPB correlated negatively with the eGFR value after ≥3 months from the operation (eGFR by CKD-EPI: r = −0.293, *p* = 0.045; eGFR by MDRD: r = −0.288, *p* = 0.049).

There was a negative correlation between urine MMP-9 concentration 6 h after weaning from CPB and postoperative kidney function after ≥3 months from the operation (MMP-9 & S_Cr_: r = 0.418, *p* = 0.003; MMP-9 & eGFR by CKD-EPI: r = −0.371, *p* = 0.010; MMP-9 & eGFR by MDRD: r = −0.301, *p* = 0.040). There was a similar correlation between urine MMP-9 concentration on the 5th day after the operation and the eGFR value after ≥3 months from the operation (MMP-9 & eGFR by CKD-EPI: r = −0.368, *p* = 0.011; MMP-9 & eGFR by MDRD: r = −0.342, *p* = 0.019).

A positive correlation was observed between urine TIMP-1 concentration 48 h after the operation and the eGFR value after ≥3 months from the operation (TIMP-1 & eGFR by CKD-EPI: r = 0.296, *p* = 0.043; TIMP-1 & eGFR by MDRD: r = 0.320, *p* = 0.028). Analogically, there was a negative correlation between the MMP-9/TIMP-1 ratio 48 h after the operation and postoperative kidney function after ≥3 months from the operation (ratio & S_Cr_: r = 0.372, *p* = 0.010; ratio & eGFR by CKD-EPI: r = −0.394, *p* =0.006; ratio & eGFR by MDRD: r = −0.365, *p* = 0.012).

### 3.3. Patients Who Developed CSA-AKI and Had Impaired Long-Term Kidney Function vs. Patients Who Developed CSA-AKI and Had Unchanged Long-Term Kidney Function

A total of 12 out of 15 patients (five women and seven men) who developed postoperative AKI made a complete recovery of kidney function after ≥3 months from the operation (follow-up eGFR > 60 mL/min/1.73 m^2^). In three female patients, long-term postoperative kidney function impairment occurred, in the form of newly diagnosed CKD.

Patients with long-term postoperative kidney function impairment after ≥3 months from the operation had higher mean hemofiltration volume (M = 1500 (1500–2000) mL vs. 250 (0–800) mL in the control group, *p* = 0.009), higher percentage of taken TBW (M = 6.01 (4.39–7.27)% vs. 0.86 (0–2.31)% in the control group, *p* = 0.009) and higher percentage of water taken from the intravascular volume (M = 47.02 (34.09–56.82)% vs. 5.43 (0–18.01)% in the control group, *p* = 0.009). Fluid balance and intraoperative diuresis did not show significant differences between the groups (*p*-value 0.448 and 0.536, respectively).

Patients who suffered from CSA-AKI and experienced long-term postoperative kidney function impairment had a significantly lower hematocrit level in the 2nd measurement during CPB (approximately 45 min after the initiation of CPB; M = 18 (18–18)% vs. 25 (22–27)% in the control group, *p* = 0.006) and a higher preoperative serum IL-8 concentration (M = 7.89 (5.25–14.85) ng/mL vs. 4.73 (3.39–5.59) ng/mL in the control group, *p* = 0.048). A higher preoperative urine IL-18 concentration was also observed in these patients (M = 36.99 (19.93–53.28) ng/mL vs. 12.48 (9.88–25.06) ng/mL in the control group), although the result lacked statistical significance (*p* = 0.070).

### 3.4. Patients Who Underwent Intraoperative Hemofiltration vs. Patients Who Did Not Undergo Intraoperative Hemofiltration

A total of 18 patients in this study population (nine women and nine men) underwent intraoperative hemofiltration, which accounts for 37.5% of this population. These patients had a lower preoperative hematocrit level (M = 37.7 (35.5–40.7)% vs. 41 (39.3–44.9)% in the control group, *p* < 0.001), a higher initial serum TNF-α concentration (M = 2.7 (2.38–3.95) ng/mL vs. 2 (1.59–2.42) ng/mL in the control group, *p* = 0.003), lower intraoperative diuresis (M = 1100 (400–1500) mL vs. 1600 (1200–2000) mL in the control group, *p* = 0.011) and higher total noradrenaline demand (M = 0.006 (0–0.009) mg/kg vs. 0 (0–0.005) mg/kg in the control group, *p* = 0.024). A positive relation between undergoing intraoperative hemofiltration and the necessity of catecholamines administration was statistically significant (OR = 3.9, CI: 1.06–14.28, *p* = 0.035). Fluid balance from the operation did not show significant differences between the groups (*p* = 0.240). Serum TNF-α concentration 6 h after weaning from CPB was higher in patients who underwent intraoperative hemofiltration (M = 5.1 (3.6–6.77) ng/mL vs. 3.98 (2.56–4.52) ng/mL in the control group, *p* = 0.020).

CSA-AKI developed in nine patients who underwent intraoperative hemofiltration (50%), compared to six patients in the control group (20%). The difference, however, did not obtain statistical significance (*p* = 0.052). The eGFR value after ≥3 months from the operation was lower in the hemofiltration group, compared to the control group (eGFR by CKD-EPI: M = 65 (58–86) mL/min/1.73 m^2^ vs. 89.5 (74–93) mL/min/1.73 m^2^ in the control group, *p* = 0.014; eGFR by MDRD: M = 67.3 (58.8–87.3) mL/min/1.73 m^2^ vs. 85.95 (75.3–96.5) mL/min/1.73 m^2^ in the control group, *p* = 0.032).

### 3.5. IL-6

Preoperative serum IL-6 concentration correlated positively with patients’ age (r = 0.419, *p* = 0.003) and the ESL value (r = 0.397, *p* = 0.005). IL-6 serum concentration 6 h after weaning from CPB was higher in patients with CSA-AKI, both in terms of absolute values (M = 128.58 (95.53–255.2) ng/mL vs. 86.62 (57.36–115.84) ng/mL in the control group, *p* = 0.005) and a percentage increase from the initial value (M = 9230 (4453.19–19551.76)% vs. 4189.89 (3001.29–7174.96)% in the control group, *p* = 0.044)—Figure 1a. Serum IL-6 concentration 6 h after weaning from CPB correlated positively with fluid balance from the operation (r = 0.473, *p* < 0.001) as well as early postoperative kidney function impairment—Table 5.

### 3.6. IL-8

Serum IL-8 concentration 6 h after weaning from CPB was higher in patients with AKI (M = 7.89 (5.25–14.85) ng/mL vs. 4.73 (3.39–5.59) ng/mL in the control group, *p* = 0.016)—Figure 1b. Moreover, preoperative serum IL-8 concentration was higher in patients with AKI in whom long-term postoperative kidney function impairment occurred, compared to patients with AKI and unchanged long-term postoperative kidney function (M = 7.89 (5.25–14.85) ng/mL vs. 4.73 (3.39–5.59) ng/mL in the control group, *p* = 0.048). Serum IL-8 concentration 6 h after weaning from CPB correlated negatively with the eGFR value during the first 72 h after the operation—Table 5.

### 3.7. TNF-α

Higher preoperative serum TNF-α concentration was observed in patients who later developed CSA-AKI (M = 2.7 (2.42–4.22) ng/mL vs. 2.13 (1.59–2.7) ng/mL in the control group, *p* = 0.012) and patients who underwent intraoperative hemofiltration (M = 2.7 (2.38–3.95) ng/mL vs. 2 (1.59–2.42) ng/mL in the control group, *p* = 0.003)—Figure 1c. There was also a correlation between preoperative serum TNF-α concentration and patients’ age (r = 0.326, *p* = 0.024), preoperative kidney function (TNF-α & S_Cr_: r = 0.290, *p* = 0.046; TNF-α & eGFR: r = −0.513, *p* < 0.001) and eGFR decline in the early postoperative period—Table 5.

Higher serum TNF-α concentration 6 h after weaning from CPB was observed in patients who developed CSA-AKI (M = 6.43 (4.5–7.08) ng/mL vs. 3.9 (2.7–4.52) ng/mL in the control group, *p* = 0.002) and in patients who underwent intraoperative hemofiltration (M = 5.1 (3.6–6.77) ng/mL vs. 3.98 (2.56–4.52) ng/mL in the control group, *p* = 0.020).

Serum TNF-α concentration 6 h after weaning from CPB correlated with patients’ age (r = 0.451, *p* = 0.001), percentage of taken TBW (r = 0.293, *p* = 0.043), percentage of water taken from the intravascular volume (r = 0.310, *p* = 0.032), mean partial oxygen pressure during CPB (r = 0.291, *p* = 0.044), serum creatinine concentration on the 1st and the 3rd postoperative day (TNF-α & S_Cr_ on the 1st day: r = 0.373, *p* = 0.009; TNF-α & S_Cr_ on the 3rd day: r = 0.295, *p* = 0.415), the preoperative eGFR value (r = −0.464, *p* < 0.001) and the eGFR value in the early postoperative period—Table 5.

### 3.8. NGAL

Urine NGAL concentration 6 h after weaning from CPB was higher in patients with CSA-AKI (M = 13231.76 (4914.81–34148.3) ng/mL vs. 4261.32 (2868.19–9138.28) ng/mL in the control group, *p* = 0.013)—Figure 1d. Urine NGAL concentration 6 h after weaning from CPB correlated with the preoperative eGFR value (r = −0.349, *p* = 0.015), diuresis from the first 24 h after the operation (r = −0.349, *p* = 0.015) and early postoperative kidney function—Table 5. The urine NGAL concentration increase 6 h after weaning from CPB correlated positively with patients’ BMI (r = 0.349, *p* = 0.015). There was a negative correlation between diuresis from the first 24 h after the operation and urine NGAL concentrations 24 h and 5 days after the operation (r = −0.323, *p* = 0.025; r = −0.319, *p* = 0.027, respectively). Persistently increased NGAL on the 5th day after the operation correlated positively with preoperative HbA1C concentration (r = 0.355, *p* = 0.015).

### 3.9. KIM-1

There were no significant differences in urine KIM-1 concentration in patients with CSA-AKI, compared to the control group—Figure 1e. There were also no differences found in urine KIM-1 concentration in patients who underwent intraoperative hemofiltration as well as patients who developed long-term postoperative kidney function impairment. Urine KIM-1 concentration, however, correlated with CPB parameters. Longer CPB time correlated positively with the percentage of KIM-1 increase at 24 and 48 h after the operation, compared to the initial value (r = 0.328, *p* = 0.023; r = 0.306, *p* = 0.035, respectively). A similar correlation was found for the aortic cross-clamp time (clamping time & percentage of KIM-1 increase at 24 h: r = 0.365, *p* = 0.011; clamping time & percentage of KIM-1 increase at 48 h: r = 0.396, *p* = 0.005).

### 3.10. IL-18

Preoperative urine IL-18 concentration was higher in patients who did not develop CSA-AKI (M = 31.05 (23.53–37.62) ng/mL vs. M = 19.93 (9.88–25.06) ng/mL in the AKI group, *p* = 0.009)—Figure 1f. Urine IL-18 concentration was higher in these patients during the whole early postoperative period (up to the 5th day after the surgery), with the exception of the 6th hour after the operation where there was no significant difference between IL-18 concentrations in both groups (*p* = 0.597). Analyzing the percentage of IL-18 increase from the initial value in the consecutive time points, it was noted that in patients with AKI, there was a significantly higher increase in IL-18 urine concentration 6 h after weaning from CPB (M = 122.02 (100–204.21)% vs. 89.80 (77.97–112.53)% in the control group, *p* = 0.002). The significance of IL-18 concentrations’ change was assessed using Friedman’s ANOVA test (*p* < 0.023) and subsequently with the Wilcoxon signed-rank test (*p* < 0.023). Friedman’s ANOVA test did not reveal any significant differences in consecutive IL-18 concentrations in the no-AKI group (*p* < 0.076). The urine IL-18 concentration in patients with AKI normalized within 48 h.

### 3.11. MMP-9

The urine MMP-9 concentration 6 h after weaning from CPB was higher in patients with CSA-AKI (M = 9661.94 (1485.62–14,451.8) ng/mL vs. 3499.39 (1274.58–8656) ng/mL in the control group, *p* = 0.044). A higher urine MMP-9 concentration correlated positively with worse preoperative kidney function (MMP-9 & S_Cr_: r = 0.430, *p* = 0.002; MMP-9 & eGFR: r = −0.368, *p* = 0.010), kidney function in the early postoperative period (Table 5) and also with kidney function after ≥3 months from the operation (MMP-9 & S_Cr_: r = 0.418, *p* = 0.003; MMP-9 & eGFR by CKD-EPI: r = −0.371, *p* = 0.010; MMP-9 & eGFR by MDRD: r = −0.301, *p* = 0.040).

Urine MMP-9 concentrations at 24 and 48 h after the operation correlated negatively with mean partial oxygen pressure during CPB (r = −0.408, *p* = 0.004; r = −0.368, *p* = 0.010, respectively). Persistently elevated MMP-9 on the 5th day after the operation correlated with the preoperative HbA1C concentration (r = 0.308, *p* = 0.037), preoperative eGFR value (r = −0.296, *p* = 0.041) and eGFR value after ≥3 months from the operation (MMP-9 & eGFR by CKD-EPI: r = −0.368, *p* = 0.011; MMP-9 & eGFR by MDRD: r = −0.342, *p* = 0.019).

### 3.12. TIMP-1

The percentage of urine TIMP-1 increase 24 h after the operation correlated positively with total CPB time and aortic cross-clamp time (r = 0.362, *p* = 0.012; r = 0.365, *p* = 0.011, respectively) as well as with intraoperative diuresis (r = 0.309, *p* = 0.032) and CRP concentrations on the 1st and the 3rd postoperative day (r = 0.298, *p* = 0.040; r = 0.419, *p* = 0.003, respectively). The urine TIMP-1 concentration 48 h after the operation was lower in patients with CSA-AKI, regarding both the absolute values (M = 1.61 (0.55–2.26) ng/mL vs. 3.3 (1.68–5.92) ng/mL in the control group, *p* = 0.007) and percentage of increase from the initial value (M = 145.05 (64.91–211.91)% vs. 268.81 (111.43–733.73)% in the control group, *p* = 0.030).

In both groups of patients (AKI and no-AKI), there was a significant increase in urine TIMP-1 concentration 24 h after the operation (AKI group: *p* = 0.003; no-AKI group: *p* = 0.037). In the AKI group, no further increase in TIMP-1 concentration was observed 48 h after the operation, unlike in the no-AKI group where there was a significant urine TIMP-1 increase (*p* < 0.001).

The urine TIMP-1 concentration 48 h after the operation correlated with the ESL value (r = −0.297, *p* = 0.040), preoperative eGFR value (r = 0.380, *p* = 0.008) and eGFR value after ≥3 months from the operation (TIMP-1 & eGFR by CKD-EPI: r = 0.296, *p* = 0.043; TIMP-1 & eGFR by MDRD: r = 0.320, *p* = 0.028). The percentage of TIMP-1 increase 48 h after the operation correlated with patients’ age (r = −0.321, *p* = 0.026) and with intraoperative diuresis (r = 0.327, *p* = 0.023).

### 3.13. MMP-9/TIMP-1 Ratio

There was no statistically significant difference between MMP-9/TIMP-1 ratio values in patients with CSA-AKI compared to the control group, at any moment of the observation. Mean partial oxygen pressure correlated negatively with MMP-9/TIMP-1 ratio values at 24 and 48 h after the operation (r = −0.472, *p* < 0.001; r = −0.318, *p* = 0.028, respectively). The MMP-9/TIMP-1 ratio value correlated positively with long-term kidney function impairment after ≥3 months from the operation (ratio & S_Cr_: r = 0.372, *p* = 0.010; ratio & eGFR by CKD-EPI: r = −0.394, *p* = 0.006; ratio & eGFR by MDRD: r = −0.365, *p* = 0.012).

### 3.14. Diuresis

It was found that intraoperative diuresis correlated with preoperative kidney function (diuresis & S_Cr_: r = −0.384, *p* = 0.007; diuresis & eGFR: r = 0.395, *p* = 0.005) and also early postoperative kidney function—Table 5. Diuresis from the operation was also significantly lower in patients who developed postoperative AKI (M = 800 (300–1500) mL vs. 1600 (1200–1900) mL in the control group, *p* = 0.006) as well as diuresis from the first 2 h after the operation (M = 300 (200–800) mL vs. 800 (400–1000) mL in the control group, *p* = 0.040). Intraoperative diuresis (adjusted for patients age) was found to be an independent predictor of CSA-AKI (OR = 0.047, CI: 0.005–0.451 over 1000 mL, *p* = 0.006). Patients who underwent intraoperative hemofiltration had lower intraoperative diuresis (M = 1100 (400–1500) mL vs. 1600 (1200–2000) mL in the control group, *p* = 0.011). Patients with long-term postoperative kidney function impairment had lower diuresis between the 2nd and 4th hour after the operation (M = 150 (75–200) mL vs. 400 (200–800) mL in the control group, *p* = 0.009).

## 4. Discussion

The main objective of this study was to assess the utility of novel kidney biomarkers in early CSA-AKI diagnostics and in the prognosis of long-term kidney function in patients after cardiac surgery procedures. The serum creatinine concentration and its derivative—eGFR are well-known and standardized kidney function indicators, validated for diagnosing AKI. Nevertheless, a long time period needed for the change of these indicators is their substantial disadvantage. A serum creatinine increase/eGFR decline happens only at the moment of advanced kidney damage [32], according to some authors even up to 48 h after CSA-AKI occurs [33].

It is consistent with the results of this study, in which most cases of CSA-AKI were diagnosed 24 h after the operation, and in some patients, a creatinine increase occurred even after 72 h. Such a delay in diagnostics and implementing the proper treatment seriously influences a patient’s prognosis [34].

It was demonstrated during the course of this study that between patients with and without CSA-AKI, significant differences exist in kidney injury biomarkers’ concentrations, as soon as 6 h after weaning from CPB. These differences mostly concern serum IL-6, IL-8 and TNF-α, urine NGAL and MMP-9 as well as the percentage of urine IL-18 increase. Among the above, independent AKI predictors proved to be IL-8 and NGAL. It is consistent with the results obtained by other authors [6,13,15,18,21,34]. Another advantage of NGAL, MMP-9 and IL-18 usage in this instant is that they are marked in urine; therefore, the test is non-invasive.

Another quality of IL-6, IL-8, TNF-α, NGAL and MMP-9, aside from indicating AKI, is that their concentrations after the operation correlate with early postoperative kidney function. For TNF-α, this correlation exists also for its preoperative value. This opens a possibility of using these biomarkers to optimize postoperative care in patients after cardiac surgery procedures.

Preoperative factors that contribute to increased risk of CSA-AKI were older age, worse initial kidney function and lower hematocrit, which is consistent with the present state of medical knowledge. Another factor that favors CSA-AKI occurrence proved to be lower intraoperative diuresis [35] and also lower diuresis during the first 2 h after the operation. Intraoperative diuresis was an independent AKI predictor, which also correlated with early postoperative kidney function. Diuresis in the early postoperative period correlated with long-term kidney function impairment. Considering that hourly diuresis assessment is non-invasive, easy-to-perform and generates very low costs, it is justified to state that intra- and postoperative diuresis measurement is a very good tool for postoperative kidney function evaluation.

Patients who developed CSA-AKI had a higher preoperative serum TNF-α concentration and a lower preoperative urine IL-18 concentration. Other authors reported that there is no connection between preoperative serum TNF-α concentration and AKI development [36,37]. Nevertheless, it was proven that serum TNF-α increases in response to prolonged inflammation related to arterial hypertension and diabetes [38] (which were present in the vast majority of patients included in this study). Another well-documented fact is that TNF-α induces inflammation in kidneys and favors their damage [39]. Considering the above, it seems logical to conclude that elevated preoperative serum TNF-α concentration reflects the subclinical proinflammatory state, which increases the risk of postoperative kidney damage.

It would be misguided to hypothesize that a higher IL-18 concentration has a protective effect on the kidneys. In patients who did not develop CSA-AKI, the postoperative IL-18 concentration maintains at a relatively unchanged level, while in patients with CSA-AKI, it rises considerably 6 h after weaning from CPB and normalizes 48 h after the operation. Similar results were obtained by other researchers [12,13]. Presumably, a relative change in IL-18 concentration compared to the initial value could be a more reliable AKI indicator than its absolute value.

Patients who did not develop CSA-AKI were statistically younger than patients with AKI, which means that they earlier developed an advanced form of heart disease that required surgical intervention. Some authors reported that patients with a genetic predisposition to a higher IL-18 concentration have a higher risk of arterial hypertension, ischemic heart disease and its complications [40,41,42,43]. This may explain the higher IL-18 phenomenon is these younger patients.

A postoperative increase in urine TIMP-1 concentration was observed in the entire study population, but it was significantly greater in patients who did not develop CSA-AKI. A considerably lower urine TIMP-1 concentration 48 h after the operation in the AKI group may reflect an imbalance between MMP-9 and TIMP-1 activities. The MMP-9/TIMP-1 imbalance is associated with multiple disorders such as autoimmune diseases, chronic obstructive pulmonary disease exacerbations and blood-brain barrier disruption [44,45,46]. Considering the above, it is justified to assume that the MMP-9/TIMP-1 imbalance may play a role in the development of CSA-AKI. Such a hypothesis is supported by a positive correlation between the MMP-9/TIMP-1 ratio value 48 h after the operation and long-term postoperative kidney function impairment observed in this study follow-up. Undermining this hypothesis, however, is the fact that there was no statistically significant difference between MMP-9/TIMP-1 ratio values 48 h after the operation in patients with AKI vs. no-AKI. Further investigation is required in this regard.

One of the factors favoring AKI progression to CKD was a higher preoperative serum IL-8 concentration. It is consistent with the findings of other authors, who proved the role of IL-8 within the kidneys in the progression of acute inflammatory disorders to the chronic form [47].

The relationship between intraoperative hemofiltration volume and long-term postoperative kidney function impairment seems to undermine the role of hemofiltration as a nephroprotective agent during CPB. The theory of protecting the kidneys by removing proinflammatory cytokines through the filtering membrane did not find any support in the results of this study. The percentage of patients with AKI was considerably greater in the hemofiltration group (borderline significance result), and the eGFR value after ≥3 months was significantly lower in these patients (though an unequivocal relationship with CKD was not proved).

There is a noticeable connection between the percentage of taken TBW and water taken from the intravascular volume and long-term postoperative kidney function impairment. It suggests that a prerenal mechanism of kidney damage secondary to hypovolemia outweighs the potential benefits coming from proinflammatory cytokines’ removal. Higher postoperative TNF-α and a higher demand for noradrenaline in the hemofiltration group both support this conclusion. It seems that a zero-balance hemofiltration could bring more benefits to the patients in this regard [48]. In light of the results of this study, intraoperative hemofiltration is a method of increasing the hematocrit level during CPB burdened with a high risk of nephrological complications.

Identification of patients endangered with long-term postoperative kidney function impairment and early implementation of the proper treatment are essential in terms of reducing morbidity and mortality among patients who had AKI [49,50]. Novel kidney injury biomarkers’ concentrations in the early postoperative period correlate significantly with serum creatinine and/or with the eGFR value ≥3 months after cardiac surgery procedure. The correlation mainly exists 6 h after weaning from CPB for TNF-α [51], MMP-9 and IL-18 [52,53], but preoperative TNF-α, TIMP-1 at 48 h, MMP-9 on the 5th day and the MMP-9/TIMP-1 ratio [54] at 48 h are also of importance here. This proves that novel kidney injury biomarkers are also an eligible tool for the assessment of CKD risk after cardiac surgery procedures.

A positive correlation between the preoperative HbA1C level and persistently elevated NGAL and MMP-9 5 days after the operation may reflect a relationship between inadequate glycemia control and prolonged inflammation after the surgery [55].

## 5. Conclusions

In conclusion, novel kidney injury biomarkers such as IL-6, IL-8, TNF-α, MMP-9 and NGAL are reliable AKI indicators, which enable early postoperative identification of patients who suffer from CSA-AKI. Serum IL-8 and urine NGAL 6 h after weaning from CPB proved to be independent AKI predictors. Another AKI predictor was intraoperative diuresis, which also strongly correlates with early postoperative kidney function. In light of the results of this study, also the preoperative serum TNF-α concentration should be considered as a possible indicator of higher AKI risk. Older age, impaired preoperative kidney function and lower perioperative hematocrit level are the most relevant factors that contribute to the occurrence of CSA-AKI. Factors favoring long-term kidney function impairment proved to be a higher preoperative serum IL-8 concentration and intraoperative hypovolemia. TNF-α, MMP-9, IL-18, TIMP-1 and the MMP-9/TIMP-1 ratio in the early postoperative period also correlated with long-term kidney function impairment. Non-zero-balanced hemofiltration did not bring any benefits in terms of protecting kidneys from CPB-related damage in this study population.

## Figures and Tables

**Table 1:** AKI diagnostic criteria according to KDIGO, AKIN and RIFLE scales.

	KDIGO	AKIN	RIFLE
S_Cr_	↑S_Cr_ ≥ 0.3 mg/dL in 48 h or ↑S_Cr_ ≥ 50% in 7 days	↑S_Cr_ ≥ 0.3 mg/dL or ↑S_Cr_ ≥ 50% in 48 h	↑S_Cr_ ≥ 50% in 7 days
Diuresis	<0.5 mL/kg/h for at least 6 h		

Legend: AKIN—AKIN kidney injury scale, KDIGO—KDIGO kidney injury scale, RIFLE—RIFLE kidney injury scale, S_Cr_—serum creatinine concentration; ↑—increase.

**Table 2:** Types of material collected from the patients and selected biomarkers of renal injury identified in them at the designated time points.

Collected Material	Time of Collection	Biomarkers Identified in the Material
serum	preoperatively(S0)	IL-6, IL-8, TNF-α
	6 h after weaning from CPB (S1)	
	6 h after finishing CRRT (S2)	
urine	preoperatively (U0)	KIM-1, NGAL, IL-18, MMP-9, TIMP-1
	6 h after weaning from CPB (U1)	KIM-1, NGAL, IL-18, MMP-9
	24 h after the operation (U2)	
	48 h after the operation (U3)	KIM-1, NGAL, IL-18, MMP-9, TIMP-1
	5 days after the operation (U4)	KIM-1, NGAL, IL-18, MMP-9

Legend: CPB—cardiopulmonary bypass, CRRT—continuous renal replacement therapy, IL-6—interleukin 6, IL-8-interleukin 8, IL—18-interleukin 18, KIM-1—kidney injury molecule 1, MMP-9—urine matrix metalloproteinase 9, NGAL—neutrophil gelatinase-associated lipocalin, TIMP-1—tissue inhibitor of metalloproteinase 1.

**Table 3:** Baseline characteristics and operation-related data of all patients initially enrolled into the study (*n* = 88).

Quality		Included (*n* = 48)	Excluded (*n* = 40)	*p*-Value
Age M, (Q1-Q3) [years]		67.5, (63–71)	70, (65–73)	0.352
Sex (*n*,%)		Female–18, 37.5	Female–13, 32.5	0.66
		Male–30, 62.5	Male–27, 67.5	
BMI M, (Q1-Q3) [kg/m^2^]		28.36, (26–31.10)	28.66, (24.95–31.63)	0.845
ESL M, (Q1-Q3) [%]		3.20, (2.16–5.1)	3.23, (2.71–6.01)	0.291
CAD (*n*,%)		I–2, 5.13	I–1, 3.45	0.557
		II–19, 48.72	II–11, 37.93	
		III–15, 38.46	III–16, 55.17	
		IV–3, 7.7	IV–1, 3.45	
HA (*n*,%)		38, 79.2	28, 70	0.337
DM (*n*,%)		12, 25	11, 27.5	0.812
HbA1C M, (Q1-Q3) [%]		5.75 (5.5–6.3)	5.9 (5.7–6.2)	0.214
Dyslipidemia (*n*,%)		12, 25	9, 22.5	0.808
CKD (*n*,%)		3a stage–5, 10.42	3a stage–4, 10	0.965
		3b stage–3, 6.25	3b stage–2, 5	
S_Cr_ 0 M, (Q1-Q3) [mg/dL]		0.91, (0.80–1.03)	0.90, (0,77–1.07)	0.655
eGFR 0 M, (Q1-Q3) [mL/min/1.73 m^2^ ]		80.5, (64.5–90.75)	82.5, (68–92)	0.643
Hematocrit 0 M, (Q1-Q3) [%]		40.3, (37.83–43.68)	41.4, (38.9–45)	0.144
CRP 0 M, (Q1-Q3) [mg/L]		1.15, (0.56–2.32)	1.35, (0.42–3.25)	0.783
CK-MB 0 M, (Q1-Q3) [IU/L]		17 (14.25–21)	16, (14–21)	0.478
IL-6 0 M, (Q1-Q3) [ng/mL]		1.66, (1.27–1.97)	X	X
IL-8 0 M, (Q1-Q3) [ng/mL]		4.39, (2.61–6.2)	X	X
TNF-α 0 M, (Q1-Q3) [ng/mL]		2.20, (1.73–3.26)	X	X
NGAL 0 M, (Q1-Q3) [ng/mL]		4512.06, (2875.93–9867.93)	X	X
KIM-1 0 M, (Q1-Q3) [ng/mL]		336.81, (122.49–992.37)	X	X
IL-18 0 M, (Q1-Q3) [ng/mL]		25.06, (18.09–36.48)	X	X
MMP-9 0 M, (Q1-Q3) [ng/mL]		135.28, (50.70–747.43)	X	X
TIMP-1 0 M, (Q1-Q3) [ng/mL]		1.01, (0.57–2.61)	X	X
MMP-9 / TIMP-1 ratio 0 M, (Q1-Q3)		150.73, (51.48–789.38)	X	X
CABG (*n*,%)		20, 41.67	23, 57.5	0.199
Valvular (*n*,%)		14, 29.17	8, 20	0.459
CABG + valvular (*n*,%)		6, 12.5	5, 12.5	1.0
Complex procedures (*n*,%)		8, 16.67	4, 10	0.535
CPB time M, (Q1-Q3) [min]		82, (67.5–105)	68, (54–110)	0.192
Aortic cross-clamp time M, (Q1-Q3) [min]		57.5, (34.75–78.75)	45, (31–65)	0.245
Exclusion criterion (*n*,%)	Catecholamines infusion	0, 0.0	33, 82.5	<0.001
	Lack of sample	0, 0.0	5, 12.5	0.024
	Infection	0, 0.0	2, 5	0.215
30-days mortality (*n*,%)		0, 0.0	1, 2.5	0.461

Legend: BMI—body mass index [kg/m^2^], CABG—coronary artery bypassing graft, CAD—coronary artery disease, CKD—chronic kidney disease, CK-MB 0—initial serum creatine kinase MB isoenzyme concentration [IU/L]; CPB time—cardiopulmonary bypass time [min], CRP 0—initial serum C-reactive protein concentration [mg/L], DM—diabetes mellitus, eGFR 0—initial estimated glomerular filtration rate [mL/min/1.73 m^2^], ESL—EuroSCORE Logistic [%], HA—hypertonia arterialis, HbA1C—glycated hemoglobin percentage [%], Hematocrit 0—initial hematocrit concentration [%], IL-6 0—initial serum interleukin 6 concentration [ng/mL], IL-8 0—initial serum interleukin 8 concentration [ng/mL], IL-18 0—initial urine interleukin 18 concentration [ng/mL], KIM-1 0—initial urine kidney injury molecule 1 concentration [ng/mL], M—median, MMP-9 0—initial urine matrix metalloproteinase 9 concentration [ng/mL], NGAL 0—initial urine neutrophil gelatinase-associated lipocalin concentration [ng/mL], S_Cr_ 0—initial serum creatinine concentration [mg/dL], TIMP-1 0—initial urine tissue inhibitor of metalloproteinase 1 concentration [ng/mL], TNF-α 0—initial serum tumor necrosis factor α concentration [ng/mL], X—given biomarker concentration/*p*-value were not available.

**Table 4:** Relationship between perioperative and long-term kidney function. Spearman rank correlation coefficients are presented together with statistical significance.

	S_Cr_ after 3 Months r (*p*–Value)	eGFR by CKD-EPI after 3 Months r (*p*–Value)	eGFR by MDRD after 3 Months r (*p*–Value)
S_Cr_ 0	0.694 *	−0.556 *	−0.569 *
eGFR 0	−0.480 *	0.773 *	0.718 *
S_Cr_ 1	0.652 *	−0.661 *	−0.686 *
eGFR 1	−0.499 *	0.745 *	0.714 *
S_Cr_ 2	0.633 *	−0.607 *	−0.586 *
eGFR 2	−0.478 *	0.737 *	0.652 *
S_Cr_ 3	0.673 *	−0.569 *	−0.546 *
eGFR 3	−0.505 *	0.745 *	0.653 *
S_Cr_ 4	0.742 *	−0.628 *	−0.631 *
eGFR 4	−0.669 *	0.820 *	0.724 *
Intraoperative diuresis	−0.393 (*p* = 0.006)	0.449 (*p* = 0.002)	0.423 (*p* = 0.003)

Legend: eGFR—estimated glomerular filtration rate [mL/min/1.73 m^2^ ], r-Spearman rank correlation coefficient, S_Cr_—serum creatinine concentration [mg/dL]; 0—initial level, 1—level from first 24 h after the operation, 2—level from the 3rd day after the operation, 3—level from the 5th day after the operation, 4—level from the 7th day after the operation; * *p*-value < 0.001.

**Table 5:** Relationship between selected parameters and early postoperative kidney function. Spearman rank correlation coefficients are presented together with statistical significance (“r” for *p*-value < 0.05 are put in bold).

	Serum IL-6 6 h after Weaning from CPB (IL6_S1) r, (*p*)	Serum IL-8 6 h after Weaning from CPB (IL8_S1) r, (*p*)	Preoperative Serum TNF-α (TNF_S0) r, (*p*)	Serum TNF-α 6 h after Weaning from CPB (TNF_S1) r, (*p*)	Urine NGAL 6 h after Weaning from CPB (NGAL_U1) r, (*p*)	Urine MMP-9 6 h after Weaning from CPB (MMP9_U1) r, (*p*)	Intraoperative Diuresis r, (*p*)
S_Cr_ 1	**0.358** (*p* = 0.013)	0.193 (*p* = 0.189)	0.247 (*p* = 0.090)	**0.373** (*p* = 0.009)	**0.343** (*p* = 0.017)	**0.380** (*p* = 0.008)	**−0.431** (*p* = 0.002)
eGFR 1	**−0.348** (*p* = 0.015)	**−0.295** (*p* = 0.042)	**−0.354** (*p* = 0.014)	**−0.455** (*p* = 0.001)	**−0.337** (*p* = 0.019)	**−0.307** (*p* = 0.034)	**0.415** (*p* = 0.003)
S_Cr_ 2	0.258 (*p* = 0.077)	0.132 (*p* = 0.369).	0.254 (*p* = 0.081)	**0.295** (*p* = 0.041)	0.262 (*p* = 0.072)	**0.470** (*p* < 0.001)	**−0.471** (*p* < 0.001)
eGFR 2	**−0.311** (*p* = 0.032)	**−0.285** (*p* = 0.050)	**−0.401** (*p* = 0.005)	**−0.418** (*p* = 0.003)	**−0.331** (*p* = 0.021)	**−0.447** (*p* = 0.001)	**0.446** (*p* = 0.001)
S_Cr_ 3	**0.304** (*p* = 0.044)	0.067 (*p* = 0.666)	0.263 (*p* = 0.085)	0.284 (*p* = 0.062)	0.209 (*p* = 0.174)	**0.461** (*p* = 0.002)	**−0.534** (*p* < 0.001)
eGFR 3	**−0.355** (*p* = 0.018)	−0.245 (*p* = 0.110)	**−0.427** (*p* = 0.004)	**−0.443** (*p* = 0.003)	**−0.327** (*p* = 0.030)	**−0.453** (*p* = 0.002)	**0.496** (*p* < 0.001)
S_Cr_ 4	0.220 (*p* = 0.219)	−0.104 (*p* = 0.564)	0.211 (*p* = 0.239)	0.202 (*p* = 0.259)	0.136 (*p* = 0.452)	**0.378** (*p* = 0.030)	**−0.471** (*p* = 0.006)
eGFR 4	−0.298 (*p* = 0.092)	0.130 (*p* = 0.472)	**−0.454** (*p* = 0.008)	**−0.420** (*p* = 0.015)	−0.244 (*p* = 0.172)	**−0.407** (*p* = 0.019)	**0.486** (*p* = 0.004)

Legend: eGFR—estimated glomerular filtration rate [mL/min/1.73 m^2^], *p*—*p*-value, r—Spearman rank correlation coefficient, S_Cr_—serum creatinine concentration [mg/dL]; 0—initial level, 1—level from first 24 h after the operation, 2—level from the 3rd day after the operation, 3—level from the 5th day after the operation, 4—level from the 7th day after the operation.

## Data Availability

The dataset generated for this study is available as Supplementary Materials.

## Supplementary Materials

The following are available online at https://www.mdpi.com/article/10.3390/biology10090823/s1, Table S1: CSA-AKI data.

## Limitations

The greatest limitation of this study was a low sample size, due to strict exclusion criteria and a significant decrease in the number of patients admitted for elective procedures caused by the COVID-19 pandemic. Another limitation was the lack of precise criteria for implementing intraoperative hemofiltration, which forced the authors to rely on a subjective assessment of the operating team.
